# Supplementary material for: Heisenberg-scaling measurement of the single-photon Kerr non-linearity using mixed states
Source: Nat Commun. 2018 Jan 8;9:93. doi: 10.1038/s41467-017-02487-z (PMC5758646; doi:10.1038/s41467-017-02487-z)
Supplement: Supplementary file 1 — Supplementary Information [file 41467_2017_2487_MOESM1_ESM.pdf]

Supplementary Note 1
Quantum Fisher information

The task at hand can be formulated as follows: We are given an evolution operator $U = e^{-igH}$, where H is a quantum mechanical operator and g is the parameter we want to estimate. In our case $H = \hat{C}\hat{n}$, where $\hat{C}$ is operating of the system and $\hat{n}$ is operating on the probe, $[\hat{n}, \hat{C}] = 0$. (Strictly speaking H is not the physical Hamiltonian, since both H and g are assumed to be dimensionless, but this form is more suitable to an information focused analysis.)

The way to estimate g would be to prepare an initial state, allow the evolution to take place and then make a final measurement on the state. We can start by analyzing what is the maximal information we could obtain from this procedure, by calculating the quantum fisher information (QFI). Consider some state, pure at this stage, $|\psi\rangle|\Psi_\mathrm{M}\rangle$, where $|\psi\rangle$ is the state of the system, related to $\hat{C}$, and $|\Psi_\mathrm{M}\rangle$ is the state of the meter, related to $\hat{n}$. The QFI is given by

$$\frac{I_\mathrm{q}}{4} = \Delta H^2 = \langle\psi|\hat{C}^2|\psi\rangle\langle\Psi_\mathrm{M}|\hat{n}^2|\Psi_\mathrm{M}\rangle - \langle\psi|\hat{C}|\psi\rangle^2\langle\Psi_\mathrm{M}|\hat{n}|\Psi_\mathrm{M}\rangle^2 = \langle\psi|\hat{C}^2|\psi\rangle\Delta n^2 + \Delta c^2\langle\Psi_\mathrm{M}|\hat{n}|\Psi_\mathrm{M}\rangle^2, \tag{1}$$

where $\Delta O^2 = \langle\hat{O}^2\rangle - \langle\hat{O}\rangle^2$ is the variance of an operator with respect to the initial state. Setting $|\psi\rangle$ to an eigenstate $\hat{C}$, with eigenvalue c would yield $I_\mathrm{q} = 4c^2\Delta n^2$, which for coherent states amounts to $I_\mathrm{q} = 4c^2N$, with N being the average photon number. For $N \gg 1$ and $\Delta c \neq 0$, the other term in I_q dominates and we have $I_\mathrm{q} \approx 4\Delta c^2N^2$. Note that this does not depend on the particular state $|\Psi_\mathrm{M}\rangle$, i.e., it is valid for coherent states just as much as it is for Fock states.

We can extend this result to mixed states by taking a weighted average of the I_q. For a statistical mixture of states $|\Psi^i_\mathrm{M}\rangle$ with probability p_i, the convexity of the QFI implies that the QFI of the mixed state, I^m_q, is bounded by $I^m_\mathrm{q} \leq \sum_i p_i I^i_\mathrm{q}$, where I^i_q is the QFI in Supplementary Eq. (1) for a state $|\Psi^i_\mathrm{M}\rangle$. That is

$$I^m_\mathrm{q} \leq 4\sum_i p_i\left(\langle\psi|\hat{C}^2|\psi\rangle\Delta_i n^2 + \Delta c^2\langle\hat{n}\rangle_i^2\right) \tag{2}$$

where $\langle\hat{n}\rangle_i = \langle\Psi^i_\mathrm{M}|\hat{n}|\Psi^i_\mathrm{M}\rangle$ is the average photon number of state $|\Psi^i_\mathrm{M}\rangle$, $\Delta_i n^2 = \langle\hat{n}^2\rangle - \langle\hat{n}\rangle^2$ is the variance for that state. In the case of coherent states, $\frac{\langle n\rangle}{\Delta n} = \sqrt{\langle n\rangle}$ so when $\langle n\rangle \gg 1$, the term containing $\langle\hat{n}\rangle_i^2$ in the QFI dominates and the bound can be approximated by:

$$4\sum_i p_i\left(\langle\psi|\hat{C}^2|\psi\rangle\Delta_i n^2 + \Delta c^2\langle\hat{n}\rangle_i^2\right) \simeq 4\sum_i p_i\Delta c^2\langle\hat{n}\rangle_i^2 = 4\Delta c^2(\mathrm{var}(n) + \bar{n}^2) \tag{3}$$

where $\mathrm{var}(n)$ and $\bar{n}$ are, respectively, the variance and average for the distribution of the photon number. We can see that by mixing coherent states one can increase the variance so that $\mathrm{var}(n) \propto N^2$, giving a bound that is $\propto 2N^2$, which improves the upper bound to the QFI. Thus, the statistical mixture does not affect how the QFI scales but it can still improve the precision.

Supplementary Note 2
Classical Fisher information

Choosing the initial state and showing that after the interaction it contain sufficient information, does not ensure that we can extract it efficiently. One should specify how to do the final measurement and show that indeed the required amount of information can be obtained. To this end, we calculate the classical Fisher information.

The particular final measurement we employed consists of post-selecting the state of the system to $|\varphi\rangle$ and measuring the number of photons in the meter.

Before the interaction the meter and the system are in a product state $\rho_0 = \rho^0_\mathrm{p}\rho^\psi_\mathrm{s}$ where ρ^0_p is the initial state of the probe and $\rho^\psi_\mathrm{s} = |\psi\rangle\langle\psi|$ is the initial state of the system. After the interaction the state evolves to

$$\rho_1 = U\rho^0_\mathrm{p}\rho^\psi_\mathrm{s}U^\dagger. \tag{4}$$

The post-selection results in an unnormalized state of the meter

$$\rho^1_\mathrm{p} = \langle\varphi|U\rho^0_\mathrm{p}\rho^\psi_\mathrm{s}U^\dagger|\varphi\rangle. \tag{5}$$

The normalization is given by $\mathcal{N} = Tr\left[\rho^1_\mathrm{p}\right] = Tr\left[U\rho^0_\mathrm{p}\rho^\psi_\mathrm{s}U^\dagger\rho^\varphi_\mathrm{s}\right]$, where $\rho^\varphi_\mathrm{s} = |\varphi\rangle\langle\varphi|$ and in the second expression the trace is over the system as well. The average photon number is given by

$$\langle\hat{n}\rangle = \mathcal{N}^{-1}Tr\left[\hat{n}\rho^1_\mathrm{p}\right] = \frac{Tr\left[\hat{n}U\rho^0_\mathrm{p}\rho^\psi_\mathrm{s}U^\dagger\rho^\varphi_\mathrm{s}\right]}{Tr\left[U\rho^0_\mathrm{p}\rho^\psi_\mathrm{s}U^\dagger\rho^\varphi_\mathrm{s}\right]}, \tag{6}$$

which is equation (4) in the main text.

Let us calculate the expression explicitly by performing the trace on Fock states $|n\rangle$ and on $\{|\varphi\rangle, |\bar{\varphi}\rangle\}$ where $|\bar{\varphi}\rangle$ is the state orthogonal to $|\varphi\rangle$. For the numerator we have

$$Tr\left[\hat{n}U\rho_{\mathrm{p}}^{0}\rho_{\mathrm{s}}^{\psi}U^{\dagger}\rho_{\mathrm{s}}^{\varphi}\right] = \sum_{n}\langle n|\langle\varphi|\hat{n}U\rho_{\mathrm{p}}^{0}\rho_{\mathrm{s}}^{\psi}U^{\dagger}|n\rangle|\varphi\rangle = \sum_{n}n\langle\varphi|e^{-ign\hat{C}}|\psi\rangle\langle n|\rho_{\mathrm{p}}^{0}|n\rangle\langle\psi|e^{ign\hat{C}}|\varphi\rangle = \sum_{n}nf_{0}(n)p_{\varphi|n} \quad (7)$$

where $f_0(n) = \langle n|\rho_{\mathrm{p}}^{0}|n\rangle$ is the initial distribution of the photon number and

$$p_{\varphi|n} = \langle\varphi|U|\psi\rangle\langle\psi|U^{\dagger}|\varphi\rangle = \langle\varphi|e^{-ig\hat{C}n}|\psi\rangle\langle\psi|e^{ig\hat{C}n}|\varphi\rangle = |\langle\varphi|\psi\rangle|^{2}(1+2g\mathrm{Im}C_{\mathrm{w}}n)+O(ng^{2}), \quad (8)$$

is the probability of post-selection for a given n, which is equation (3) in the main text. We assume that $|C_{\mathrm{w}}|ng \ll 1$ where $f_0(n)$ has significant support so we neglect from now all $O(ng^2)$ terms (in the next section we examine this assumption more carefully).

The calculation for the denominator is similar (identical besides the factor of n) and inserting the two expressions into Supplementary Eq. (6) we get

$$\langle\hat{n}\rangle \simeq \frac{\sum_{n}f_{0}(n)n(1+2g\mathrm{Im}C_{\mathrm{w}}n)}{\sum_{n}f_{0}(n)(1+2g\mathrm{Im}C_{\mathrm{w}}n)} = \frac{\langle n\rangle_{0}+2g\mathrm{Im}C_{\mathrm{w}}\langle n^{2}\rangle_{0}}{1+2g\mathrm{Im}C_{\mathrm{w}}\langle n\rangle_{0}} \simeq N+2\frac{g}{\varepsilon}(\Delta n)^{2}, \quad (9)$$

where $N = \langle n\rangle_0$ and $(\Delta n)^2 = \langle n^2\rangle_0 - \langle n\rangle_0^2$ are the initial average and variance of the photon number, respectively and $\langle\dot{}\rangle_0$ is an average over the $f_0(n)$. This gives us equation (5) in the main text. We can also see that the particular form of $f_0(n)$ does not matter, the only important quantities are its first and second moments (mean and variance).

The classical Fisher information can be obtained from $f(n,g) = f_0(n)p_{\varphi|n}$, the modified distribution of the photon number after the interaction and post-selection, which depends on g. The change $f_0(n) \to f(n,g)$ is due to the dependency of the post-selection probability on n following the interaction. Using Supplementary Eq. (8) we have

$$f(n,g) = f_{0}(n)p_{\phi|n} = \frac{f_{0}(n)(1+2g\mathrm{Im}C_{\mathrm{w}}n)}{\sum_{n'}f_{0}(n')(1+2g\mathrm{Im}C_{\mathrm{w}}n')} = \frac{f_{0}(n)(1+2g\mathrm{Im}C_{\mathrm{w}}n)}{1+2g\mathrm{Im}C_{\mathrm{w}}\langle n\rangle_{0}} = f_{0}(n)(1+2g\mathrm{Im}C_{\mathrm{w}}(n-\langle n\rangle_{0})), \quad (10)$$

where in the last step we used $\frac{1}{1+x} \simeq 1-x$. The Fisher information is given by $FI = \langle\left(\frac{\partial \ln f(n,g)}{\partial g}\right)^2\rangle$. Taking the logarithm and differentiation yields

$$\frac{\partial \ln f(n,g)}{\partial g} = \frac{2\mathrm{Im}C_{\mathrm{w}}(n-\langle n\rangle_{0})}{1+2g\mathrm{Im}C_{\mathrm{w}}(n-\langle n\rangle_{0})}. \quad (11)$$

After we differentiated with respect to g, we can take just the zero order in g, i.e., ignore the denominator above. The Fisher information is then

$$I_{\mathrm{c}}^{1} = 4\mathrm{Im}C_{\mathrm{w}}^{2}\langle(n-\langle n\rangle_{0})^{2}\rangle = 4\mathrm{Im}C_{\mathrm{w}}^{2}\Delta n^{2}. \quad (12)$$

For a distribution such that $\Delta n \propto N$, this results scales as N^2, the same as in Supplementary Eq. (3). Note that this is the CFI per postselected run. The prefactor $\mathrm{Im}C_{\mathrm{w}}^2$ will be compensated by the postselection probability, as we show below.

Supplementary Note 3
Weak measurement

We present our scheme using the formalism of Weak Measurement, which involves some controversy in the literature. In our scheme it includes two issues: the small parameter approximation and the use of post-selection. Before elaborating on these, let us just emphasize that the formalism is not necessary to understand our experiment. The main reason for using it is that it gives us a rather simple picture, using reasonable analytic expressions, which can greatly assist in designing experimental setups.

Small parameter approximation Formally our method requires $f_0(n)$ to have significant support only where $|C_{\mathrm{w}}|ng \ll 1$, and in particular where $\eta \equiv Ng/\varepsilon \ll 1$. Since, in general, the precision improves as N is increased we should analyze the situation for higher values of η. In the experiment values up to $\eta \simeq 3$ were probed (technical limitations, mainly due to the filtering of the probe, hindered us from going to larger values). We also performed numerical calculations, shown in Supplementary Figure 1. The deviation from the Heisenberg scaling start being significant around $\eta \simeq 0.1$, and around $\eta \simeq 10$ the precision reaches a maximum. Increasing N beyond this point would decrease the precision.

As long as the limit on N is such that the relevant regime is $\eta \ll 1$, the Heisenberg scaling is clearly demonstrated. In our scheme when $g \to g = a^{-1}g$ and $N \to N = aN$, the relative precision $\frac{g}{\Delta g}$ does not change, for any number $a > 0$. This fact

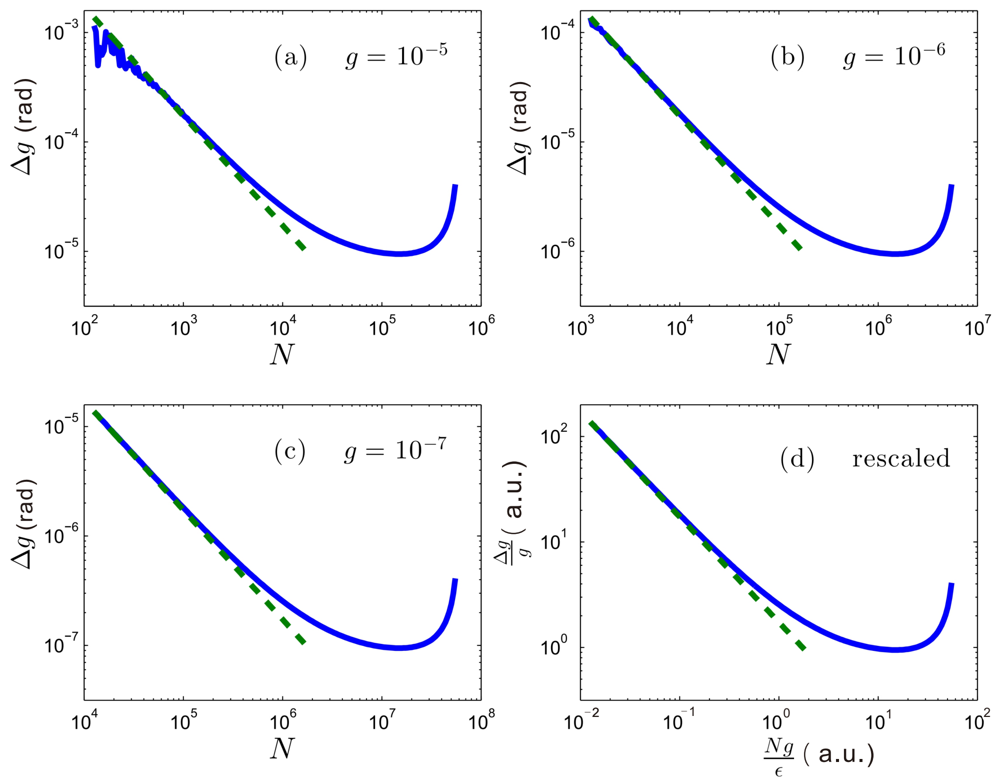

Supplementary Figure 1. Numerical calculation beyond the Weak Measurement approximation. The calculation was done according to the the description in the text, with $\varepsilon = 0.1$. Panels (a)-(c) are showing the precision Δg as function of the photon number N, calculated for different values of g. (d) Rescaling of the precision and the photon number: The plot is showing the relative precision $\frac{\Delta g}{g}$ as a function of our small parameter $\eta \equiv Ng/\varepsilon$. Doing this for any of the plots (a)-(c) results in exactly same curve. The dashed line in all the plots corresponds to the Heisenberg scaling and is given by $\Delta g^{-1} = \frac{2}{\varepsilon}\frac{N}{\sqrt{3}} = \sqrt{I_c^1}$. For the highest value of g and lower values of η the discretization of the photon number created some numerical imperfections, shown at the upper left corner.

can be analytically deduced from the Hamiltonian, and we also demonstrate it in the numerical calculations shown in shown in Supplementary Figure 1.

We preformed numerical calculations by taking the pre- and postselected states to be $|\psi\rangle = (|\uparrow\rangle + |\downarrow\rangle)/\sqrt{2}$ and $|\varphi\rangle = (|\uparrow\rangle - e^{i\varepsilon}|\downarrow\rangle)/\sqrt{2}$, where $|\uparrow\rangle$ ($|\downarrow\rangle$) is an eigenstate of $\hat{C}$, with eigenvalue 1 (0). The probability in Supplementary Eq. (8) was replaced by the exact expression $p_{\varphi|n} = \left|\langle\varphi| e^{-ig\hat{C}\hat{n}} |\psi\rangle\right|^2 = \frac{1}{2}(1-\cos(gn+\varepsilon))$. The sum in Supplementary Eq. (7) was done numerically using a uniform distribution $f_0(n) = \frac{1}{2N}$ for $n < 2N$, having $\langle n\rangle_0 = N$ and $\Delta n^2 = \frac{N^2}{3}$. We plotted the relative precision $\frac{\Delta g}{g} = \frac{\Delta n}{\delta n}$ for a few order of magnitudes of N and g.

Post-selection As we emphasized in the main text, the post-selection in this scheme does not affect the scaling behavior since the portion of post-selected runs, $|\langle\varphi|\psi\rangle|^2$, is largely independent of N. Moreover, our scheme is not based on the so-called Weak Value amplification, in the sense that the factor coming from a large Weak Value is not significant for the major improvement in precision.

We can analyze this effect by looking on I_c in Supplementary Eq. (12). Information is additive, and since I_c^1 is calculated for a single-run of the experiment, one should multiply by the number of runs $\tilde{\nu}$. We consider only runs that are post-selected. The

frequency of post-selection is $\simeq |\langle\phi|\psi\rangle|^2$ so the total Fisher information is

$$I_{\rm c} = 4\tilde{\nu}{\rm Im}C_{\rm w}^2\,|\langle\phi|\psi\rangle|^2\,\Delta n^2 \simeq \tilde{\nu}\Delta n^2. \tag{13}$$

The pre-factor $4{\rm Im}C_{\rm w}^2\,|\langle\phi|\psi\rangle|^2 \simeq 1$ does not depend on N. Its exact value, and the values ${\rm Im}C_{\rm w}$ and $|\langle\phi|\psi\rangle|^2$ are determined by the details of the experiment. The optimal setting depends on practical considerations, such as efficiency of the different detectors, data acquisition etc.

Supplementary Note 4
The implementation in a Ramsey-type model with qubits

In this section we present a detailed derivation of the scheme for the case in which the system and probes are qubits. The purpose here is to describe the scheme in, perhaps, a more general context, and to provide an alternative detailed derivation of the scheme.

The interaction Hamiltonian between a single spin (system) and an ensemble of other N spins (probes) is given by $H = f(t)\sigma_z S_z$, where σ_z ($S_z = \sum_{i=1}^N \sigma_z^i$) is operating on the system (probes). The objective is to estimate $g = \int f(t)dt$ by a measurement of the probes after initialization and interaction. Consider first an initial pure product state of the probes. Due to the coherent interaction between the system and the probes, the QFI of the joint system-probes state is $\propto N^2$. This could be obtained, for example, by initializing all probes to the same σ_z eigenstate and measuring the single spin, which acquires a phase $\propto gN$. However, if instead one measures the phase that the probes accumulate due to the interaction, then the estimation of g is limited by the SQL; each probe acquires a phase $\propto g$. Of course, an initial maximally entangled state of the probes can yield (assuming no decoherence) an Heisenberg limited estimation, $\Delta g \propto 1/N$, because in this case the phase acquired by the probes is $\propto gN$.

In our method, however, we utilize a mixed state of the probes together with a post-selection of the system in such a way that a precision with a scaling of $\Delta g \propto 1/N$ is obtained for a measurement of S_z of the probes (in this case a population rather than a phase measurement).

Denote the initial mixed state of the probes by $\rho_{\rm p} = \sum_a p_a |\Psi_a\rangle\langle\Psi_a|$, where p_a is the probability of having an initial product state $|\Psi_a\rangle$ for the ensemble of spins. The single spin is prepared in a state $|\psi\rangle$ and after the interaction is post-selected to $|\varphi\rangle$. By first considering a single pure product state of the probes, $|\Psi_a\rangle$, and assuming that $gN \ll 1$, the interaction results in

$$|\chi\rangle \simeq (\mathbb{I} - ig\sigma_z \otimes S_z)\,|\psi\rangle\,|\Psi_a\rangle\,, \tag{14}$$

and the post-selection probability is calculated as

$$\begin{aligned} p_{\varphi|a} &= Tr(|\varphi\rangle\langle\varphi| \otimes \mathbb{I}_{\rm p}\,|\chi\rangle\langle\chi|) \\ &= |\langle\varphi|\psi\rangle|^2\,(1 + 2g{\rm Im}(\sigma_z)_w\,\langle\Psi_a|\,S_z\,|\Psi_a\rangle) + o(g^2), \end{aligned} \tag{15}$$

where $\mathbb{I}_{\rm p}$ is the identity matrix operating on the probes' subspace, and $(\sigma_z)_{\rm w} = \frac{\langle\varphi|\sigma_z|\psi\rangle}{\langle\varphi|\psi\rangle}$ is the weak value of σ_z. The corresponding final state of the probes is given by

$$\begin{aligned} |\Psi_a^f\rangle &\simeq \frac{1}{\sqrt{p_{\varphi|a}}}\langle\varphi|\chi\rangle \\ &= \frac{1}{\sqrt{p_{\varphi|a}}}\Big(\langle\varphi|\left(\mathbb{I} - ig\sigma_z \otimes S_z\right)|\psi\rangle\Big)\,|\Psi_a\rangle \\ &= \frac{1}{\sqrt{p_{\varphi|a}}}\Big(\langle\varphi|\psi\rangle - ig\,\langle\varphi|\,\sigma_z\,|\psi\rangle\,S_z\Big)\,|\Psi_a\rangle \\ &= \frac{\langle\varphi|\psi\rangle}{\sqrt{p_{\varphi|a}}}\Big(\mathbb{I} - ig(\sigma_z)_{\rm w}S_z\Big)\,|\Psi_a\rangle \\ &\simeq \frac{\langle\varphi|\psi\rangle}{\sqrt{p_{\varphi|a}}}e^{-ig(\sigma_z)_{\rm w}S_z}\,|\Psi_a\rangle\,. \end{aligned} \tag{16}$$

For the initial mixed state of the probes, $\rho_{\rm p}$, the interaction leads to

$$\rho \simeq (\mathbb{I} - ig\sigma_z \otimes S_z)\,|\psi\rangle\langle\psi| \otimes \rho_{\rm p}(\mathbb{I} + ig\sigma_z \otimes S_z), \tag{17}$$

and the post-selection results in the final state of the probes

$$\rho_{\rm p}^f \simeq \frac{1}{N_\rho}\sum_a p_a p_{\varphi|a}\,|\Psi_a^f\rangle\langle\Psi_a^f|\,, \tag{18}$$

where $N_\rho = \sum_a p_a p_{\varphi|a}$ is the re-normalization due to the post-selection probability. We choose $|\psi\rangle$ and $|\varphi\rangle$ such that $(\sigma_z)_\mathrm{w}$ is mainly imaginary. Then the observable S_z, appearing in the Hamiltonian, is measured, resulting in

$$\begin{aligned}\langle S_z\rangle &= Tr[\rho_\mathrm{p}^f S_z]\\ &\simeq \frac{1}{N_\rho} Tr\left[\left(\sum_a p_a |\langle\varphi|\psi\rangle|^2 (\mathbb{I} + g\mathrm{Im}(\sigma_z)_\mathrm{w}\otimes S_z)|\Psi_a\rangle\langle\Psi_a|(\mathbb{I}+g\mathrm{Im}(\sigma_z)_\mathrm{w}\otimes S_z)\right)S_z\right].\end{aligned} \tag{19}$$

Keeping only terms of first order in g we have that

$$\langle S_z\rangle \simeq \frac{1}{N_\rho}\sum_a p_a |\langle\varphi|\psi\rangle|^2 \left(\langle\Psi_a|S_z|\Psi_a\rangle + 2g\mathrm{Im}(\sigma_z)_\mathrm{w}\langle\Psi_a|S_z^2|\Psi_a\rangle\right) \tag{20}$$

Substituting the expressions of $p_{\varphi|a}$ and N_a, and keeping only terms of first order in g, we conclude that

$$\begin{aligned}\langle S_z\rangle &\simeq \frac{\sum_a p_a |\langle\varphi|\psi\rangle|^2 \left(\langle\Psi_a|S_z|\Psi_a\rangle + 2g\mathrm{Im}(\sigma_z)_\mathrm{w}\langle\Psi_a|S_z^2|\Psi_a\rangle\right)}{\sum_a p_a |\langle\varphi|\psi\rangle|^2 (1+2g\mathrm{Im}(\sigma_z)_\mathrm{w}\langle\Psi_a|S_z|\Psi_a\rangle)}\\ &= \frac{\langle S_z\rangle_0 + 2g\mathrm{Im}(\sigma_z)_\mathrm{w}\langle S_z^2\rangle_0}{1+2g\mathrm{Im}\sigma_\mathrm{w}\langle S_z\rangle_0}\\ &\simeq S_{z0} + 2g\mathrm{Im}(\sigma_z)_\mathrm{w}\left(\langle S_z^2\rangle_0 - \langle S_z\rangle_0^2\right),\end{aligned} \tag{21}$$

where $\langle\bullet\rangle_0$ means an average with respect to ρ_p before the interaction. For the modified state $\langle S_z\rangle \simeq \langle S_z\rangle_0 + 2g\mathrm{Im}(\sigma_z)_\mathrm{w}\left(\langle S_z^2\rangle_0 - \langle S_z\rangle_0^2\right)$. This yields an estimation for g with a precision of $\Delta g = \frac{\Delta\langle S_z\rangle}{|\frac{d\langle S_z\rangle}{dg}|} \sim \frac{1}{\Delta H}$. By preparing a mixed state with $\Delta H \propto N$, for example, $\rho = \frac{1}{2}\left(\prod_{i=1}^N |\uparrow_z^i\rangle\langle\uparrow_z^i| + \prod_{i=1}^N |\downarrow_z^i\rangle\langle\downarrow_z^i|\right)$, an Heisenberg-like scaling is achieved.

This scheme may prove to be useful in case that there is some type of asymmetry between the system spin and the probe spins due to which, it is preferable to measure the probes.

Supplementary Note 5
Experimentally quantification of the precision

A major part of our results is a demonstration that the relevant precision was achieved in practice. In this context, calculation the Fisher information is not enough since it only gives us a bound. We want to see directly how sensitive is our method to changes in g. The quantity that is directly read out from the experimental equipment is $\delta\tilde{n}$. Its uncertainty $\Delta\tilde{n} = \sigma/\sqrt{\nu}$ is also determined by this equipment. The precision of our method is then given by

$$\Delta g = 2\left(\frac{\partial\delta\tilde{n}}{\partial g}\right)^{-1}\Delta\tilde{n}, \tag{22}$$

which is the quantity plotted in Fig. 4 in the main text. In order to obtain $\frac{\partial\delta\tilde{n}}{\partial g}$, we modify g artificially, by tuning the overlap. The results are shown in Supplementary Figure 2.

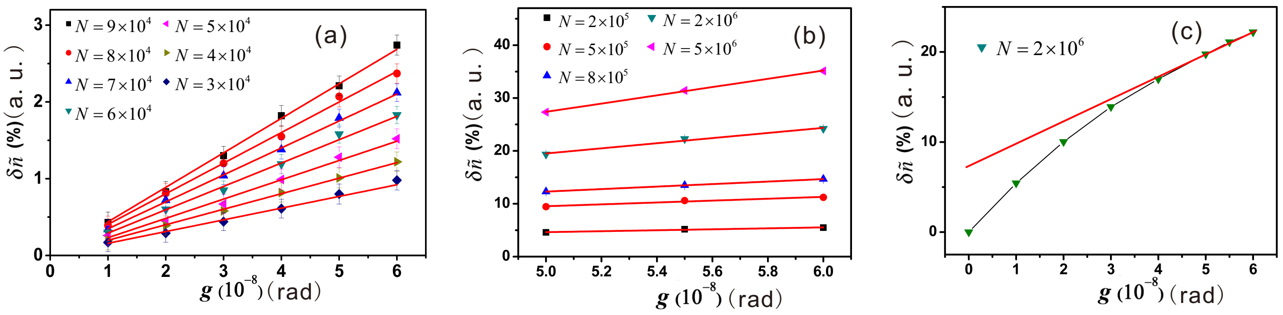

Supplementary Figure 2. Calculation of measurement precision. The normalized change in the photon number $\delta\tilde{n}$ is measured for a number of values of N by varying interaction parameter g, via tuning the temporal overlap of the system and probe, with $\varepsilon = 0.1$, $\Delta n \simeq 0.5N$ and $\nu \simeq 2.2\times10^5$. $s = \frac{\partial\delta\tilde{n}}{\partial g}$ is given by the slope of the linear fitting slope (shown as solid lines) and $\Delta\tilde{n}$ is given by the error bars (which are smaller than the marker size in panel (b) and (c)). Their ratio $\Delta g = \frac{2\Delta\tilde{n}}{s}$ is plotter in Fig. 4 in the main text. Panel (a) shows the regime of $Ng/\varepsilon \ll 1$, where the $s \propto N$. Since $\Delta\tilde{n}$ is roughly independent of N this yields the Heisenberg scaling. The fitting yields $s = (1.52, 2.03, 2.4, 3.04, 3.51, 3.97, 4.48) * 10^5$. Panel (b): for higher value of N, the slope is not linearly proportional to N anymore and a deviation from the Heisenberg scaling is observed. The fitting yields $s = (9.02, 17.7, 23.7, 48.4, 78.1) * 10^5$. Panel (c): When Ng/ε is not small, $\delta\tilde{n}$ is not linear in g. The slope s is given by a linear fit at the maximal g, as shown to be the red line in panel (c). The error bars are shown as the uncertainty in $\delta\tilde{n}$, which is written as $\sigma/\sqrt{\nu}$. Here, σ is the standard deviation of measured $\delta\tilde{n}$ and ν is number of recorded probe pulses by FHO.
